# Prior Treatment with AICAR Causes the Selective Phosphorylation of mTOR Substrates in C2C12 Cells

**DOI:** 10.3390/cimb45100508

**Published:** 2023-09-30

**Authors:** Cass J. Dedert, Kazimir R. Bagdady, Jonathan S. Fisher

**Affiliations:** Department of Biology, Saint Louis University, St. Louis, MO 63103, USA

**Keywords:** unc-like kinase 1, mammalian target of rapamycin, mechanistic target of rapamycin, 5-aminoimidazole-4-carboxamide ribonucleoside, C2C12 cells

## Abstract

Metabolic stress in skeletal muscle cells causes sustained metabolic changes, but the mechanisms of the prolonged effects are not fully known. In this study, we tested C2C12 cells with the AMP-activated protein kinase (AMPK) stimulator AICAR and measured the changes in the metabolic pathways and signaling kinases. AICAR caused an acute increase in the phosphorylation of the AMPK target ULK1, the mTORC1 substrate S6K, and the mTORC2 target Akt. Intriguingly, prior exposure to AICAR only decreased glucose-6 phosphate dehydrogenase activity when it underwent three-hour recovery after exposure to AICAR in a bicarbonate buffer containing glucose (KHB) instead of Dulbecco’s Minimum Essential Medium (DMEM). The phosphorylation of the mTORC1 target S6K was increased after recovery in DMEM but not KHB, although this appeared to be specific to S6K, as the phosphorylation of the mTORC1 target site on ULK1 was not altered when the cells recovered in DMEM. The phosphorylation of mTORC2 target sites was also heterogenous under these conditions, with Akt increasing at serine 473 while other targets (SGK1 and PKCα) were unaffected. The exposure of cells to rapamycin (an mTORC1 inhibitor) and PP242 (an inhibitor of both mTOR complexes) revealed the differential phosphorylation of mTORC2 substrates. Taken together, the data suggest that prior exposure to AICAR causes the selective phosphorylation of mTOR substrates, even after prolonged recovery in a nutrient-replete medium.

## 1. Introduction

The drug 5-aminoimidazole-4-carboxamide ribonucleoside (AICAR) has long been used in vitro as an exercise mimetic due to its metabolism into the AMP analog ZMP following cellular uptake [1]. Traditional studies using this treatment modality have emphasized the activation of AMP-activated protein kinase (AMPK) downstream of AICAR treatment, since AMPK is activated by an increase in the AMP/ATP ratio [2]. This is of particular interest in skeletal muscle, in which AICAR has substantiated a link between AMPK activation and increased fatty acid oxidation [3], glucose uptake [4], and the suppression of protein synthesis via the inhibition of the mammalian/mechanistic target of rapamycin (mTOR) [5]. However, treatment with AICAR has been demonstrated to cause changes in signaling even in AMPK-knockout models, suggesting that its effects extend beyond the AMPK signaling pathways [6].

mTOR, in contrast to AMPK, responds to conditions of nutrient sufficiency and influences metabolism through two distinct complexes, mTORC1 and mTORC2 [7]. mTORC1 is activated by insulin [8], the availability of cytosolic or lysosomal amino acids [7], mechanical signals during muscle contraction [9], and reciprocally when AMPK activity declines [2]. mTORC1 initiates anabolic pathways such as protein synthesis [7]. mTORC2 mediates insulin signaling as an activating kinase for Akt [10,11], which it phosphorylates when mTORC2 autoinhibition ceases in response to insulin’s activation of phosphatidylinositol-3 kinase (PI3K) [7]. Thus, mTORC2, via its participation in insulin signaling, is an upstream activator of mTORC1.

After metabolic stress in skeletal muscle, prolonged metabolic effects linger. For example, insulin sensitivity is increased for hours after either exposure to AICAR or a set of 10 maximal skeletal muscle contractions [12]. Likewise, the phosphorylation of the mTORC1 effector S6K is higher several hours after muscle contractions than it is immediately after exercise, and it remains elevated for at least 36 h [13]. However, AMPK activation returns to baseline within several hours of exercise or AICAR treatment [14,15], leading to uncertainty about what events downstream of AICAR treatment might promote these effects.

Given the evidence of sustained metabolic signals after metabolic stress and the interplay between AICAR, mTOR, and AMPK, the goal of this study was to elucidate the prolonged changes in the phosphorylation of mTORC1 and mTORC2 substrates after the exposure of C2C12 cells to AICAR. We hypothesized that AICAR-mediated kinase activation results in a unique phosphorylation profile involving mTOR substrates despite the conflicting roles of AMPK and mTORC1. C1C12 mouse myoblasts were differentiated for 48 h and subsequently tested to mimic the effects of AICAR on myofibers in an in vitro model. Our results indicated that certain downstream substrates of mTORC1/2 are affected while others remain unchanged, suggesting that AICAR-mediated activation causes the heterogenous phosphorylation of mTOR substrates in skeletal muscle cells.

## 2. Materials and Methods

### 2.1. Reagents

Unless otherwise specified, reagents were purchased from Sigma (St. Louis, MO, USA). The following listed antibodies were purchased from Cell Signaling Technologies (Danvers, MA, USA): p-AMPKα-thr172 (2531S), AMPKα (2532S), p-ULK1-ser555 (5869S), p-ULK1-ser757 (6888S), ULK1 (8054S), GAPDH, HRP-conjugated (8884S), p-S6K-thr389 (9205S), S6K (9202S), p-Akt-ser473 (9271S), Akt (9272S), p-PKCα-thr638 (9375S), PKCα (2056S), p-Akt substrate (6950S), p-GSK3β-ser9 (5558S), and GSK3β (9315S). The antibodies for pSGK1-ser422 (Cat# 44-1264G) and SGK1 (Cat# PA5-21147) were purchased from Invitrogen (Carlsbad, CA, USA). DMEM and PBS were purchased from Thermo Fisher (Waltham, MA, USA).

### 2.2. Cells Studied

The C2C12 mouse myoblast cells were purchased from ATCC (Manassas, VA, USA). The cell culture procedures were generally as described by Somwar et al. [16], with slight modifications [17]. The cells were grown in Delbecco’s modified Eagle Medium (DMEM, Thermo Fisher) supplemented with 10% FetalPlex (Gemini Bio Sciences, Sacramento, CA, USA) and 1% penicillin/streptomycin solution (Sigma). The cells were incubated at 37 ℃ at 5% CO_2_ and passaged every 48–72 h.

### 2.3. Cell Plating and Harvest

The C2C12 cells were grown to confluence in a 12-well plate before incubation in DMEM with 2% horse serum (Sigma) and 1% pen/strep for 24–48 h in a 37 ℃, 5% CO_2_ environment for differentiation. Myoblast differentiation was verified by observation using a phase-contrast microscope (IX73, Olympus), and the cells were in differentiation medium for 2–3 days before the experiments, which were initiated only after the cultures developed an elongated morphology associated with differentiation. Afterwards, the cells were incubated in DMEM or Krebs–Henseleit Bicarbonate (KHB) buffer (pH 7.2–7.4) [12] supplemented with 5 mM of glucose. For the experiments with AICAR, the cells were incubated with 2 mM of AICAR in DMEM for 60 min.

After treatment, the cells were rinsed and incubated in fresh DMEM or KHB with 5 mM of glucose for 3 h. The cells were scraped in a lysis buffer containing phosphatase and protease inhibitors (50 mM of HEPES, pH 7.4, 150 mM of NaCl, 10% glycerol, 1% Triton X-100, 1.5 mM of MgCl_2_, 1 mM of EDTA, 10 mM of sodium pyrophosphate, 100 mM of NaF, 2 mM of Na_3_VO_4_, 10 μg/mL of aprotinin, 10 μg/mL of leupeptin, 0.5 μg/mL of pepstatin, and 0.2 mM of PMSF) and centrifuged at 14,000 rpm for 10 min, and the supernatant was saved [18].

### 2.4. Enzymatic Activity Assay

The protein concentration of the sample lysates was measured using the bicinchoninic acid method (Thermo Fisher Pierce BCA protein assay), as per the manufacturer’s directions. In each enzyme activity assay, the activity was measured using the linear portion of the curve of the absorbance change at 340 nm for NADPH. The reaction-specific rate was calculated by subtracting sample blanks from the samples containing substrate. The activity of glucose-6-phosphate dehydrogenase (G6PD), phosphofructokinase (PFK), and fructose-1,6-bisphosphatase (FBP1) was measured as in Passoneau and Lowry [19].

For G6PD activity, a reagent medium containing 100 mM of glycine (pH 9.4), 0.5 mM of NADP^+^, 0.5 M of EDTA, and 0.02% BSA was used. The absorbance change was recorded before and after the addition of 2 mM of glucose-6-phosphate.

For PFK activity, a reagent medium containing 50 mM of Tris-HCl (pH 8.1), 1 mM of AMP, 10 mM of K_2_HPO_4_, 1 mM of DTT, 2 mM of MgCl_2_, 0.1 mM of NADH, 1 U/mL of aldolase, 5 U/mL of G6PDH, and 15 U/mL of triosephosphate isomerase was used. The absorbance change was recorded before and after the addition of 1 mM of fructose-6-phosphate.

For FBP1 activity, a reagent medium containing 50 mM of Tris-HCl (pH 7.0), 100 mM of EDTA, 0.05% BSA, 2 mM of DTT, 2 mM of MgCl_2_, 0.1 mM of NADP^+^, 0.25 U/mL of phosphoglucoisomerase, and 0.18 U/mL of G6PDH was used. The absorbance change was recorded before and after the addition of 0.4 mM of fructose-1,6-bisphosphate. 

### 2.5. Western Blot

The samples were run on 4–20% tris-glycine SDS-polyacrylamide gels (Expedeon, Cambridge, MA, USA) and transferred to 0.1 µm pore size nitrocellulose membranes for blotting. To determine the molecular weight of the target proteins, a pre-stained protein ladder (Bullseye, MidSci, Cat# BEPAR) was loaded alongside the samples. The membranes were incubated overnight in a 1:1000 concentration of primary antibody in 1% milk in TRIS-buffed saline with tween (TBST). The membranes were then incubated in a 1:5000 concentration of secondary antibody for one hour before washing with TBST, followed by incubation with Western Lightning enhanced chemiluminescence reagent (Perkin Elmer, Waltham, MA) and imaging (iBright FL2000, Thermo Fisher). After the imaging, the membranes were stripped by washing with 0.2 N NaOH twice for five minutes each time, followed by washing with TBST for ten minutes twice each time.

A Western blot densitometric analysis was performed using ImageJ software (NIH). The level of protein expression was calculated as a ratio of the band intensity for the phosphorylated protein to total protein, or to a loading control (GAPDH) as appropriate. The intensity values were normalized, with the control equal to 1.0.

### 2.6. Statistics

Data are expressed as mean ± standard error of the mean (SEM). Statistical significance was tested in SPSS Statistics 25 (IBM) with an alpha value of 0.05. For the comparison of two groups, Student’s *t*-test was used. For the comparison of more than two groups, one- or two-way ANOVA was used. Fisher’s LSD was used for *post hoc* testing.

## 3. Results

**Figure 1:**
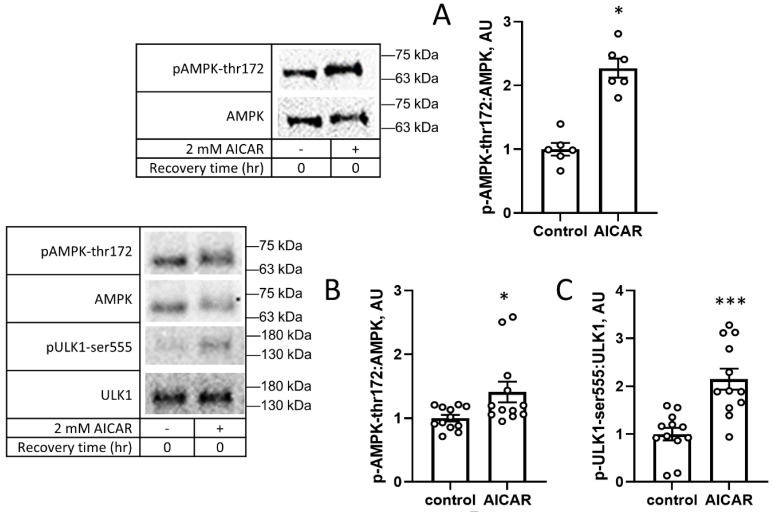
The AMP analog AICAR increases AMPK and ULK1 phosphorylation. C2C12 cells were treated with 2 mM AICAR for 1 h, then immediately lysed and harvested for Western blot analysis. (**A**,**B**) Two experimental replicates of AMPK phosphorylation immediately after incubation with AICAR, and (**C**) ULK1 phosphorylation at the AMPK target, serine 555 (pULK1-ser555) immediately after incubation with AICAR. Values are means ± SEMs. N = 12/group (N = 6/group for A). *, *p* < 0.05; ***, *p* < 0.001. AU: arbitrary units.

One hour of incubation of the C2C12 cells with the AMP analog AICAR was sufficient for increasing the AMPK phosphorylation at its activating thr172 site, which we validated through repeat experimentation (Figure 1A,B). Immediately after treatment with AICAR, ULK1 phosphorylation at its AMPK target site, ser555, was increased as well (Figure 1C), demonstrating the functional activation of AMPK by AICAR in these cells. These data indicate that exposure to AICAR increased the phosphorylation of ULK1, a key promoter of autophagy induction.

**Figure 2:**
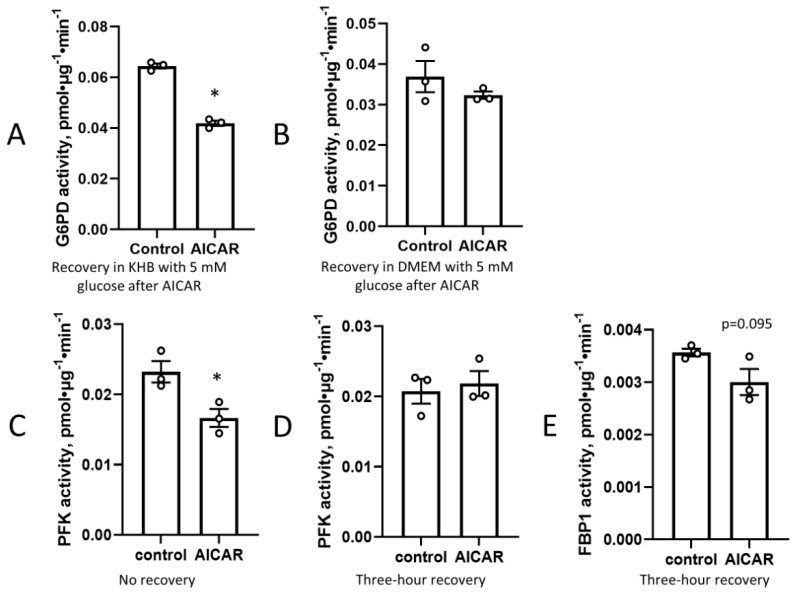
Presence of nutrients during recovery blunts the effect of AICAR on metabolic enzyme activity. C2C12 cells were incubated with 2 mM AICAR for 1 h, then incubated in AICAR-free (**A**) KHB with 5 mM glucose or (**B**) DMEM for three hours before cells were harvested and assayed for G6PD activity. Phosphofructokinase (PFK) activity decreased (**C**) immediately after 1 h AICAR treatment, but was not significantly different (**D**) after a three-hour recovery period in DMEM. (**E**) Fructose-1,6-bisphosphatase (FBP1) activity was not significantly affected by AICAR after a three-hour recovery period in DMEM. Values are means ± SEMs. *n* = 3/group. *, *p* < 0.05.

It has previously been reported that the activation of AMPK causes a decrease in the expression of glucose-6 phosphate dehydrogenase (G6PD) [20], as well as a decrease in glycolytic activity [21,22]. We found that G6PD activity decreased after prior exposure to AICAR followed by 3 h recovery in KHB containing 5 mM of glucose (Figure 2A). Strikingly, the effect of prior exposure to AICAR was prevented when the cells recovered in DMEM instead of KHB (Figure 2B). A similar phenomenon was seen in the activity of phosphofructokinase (PFK) and fructose-1,6,-bisphosphatase (FBP1), two rate-limiting enzymes of glycolysis. Three-hour recovery in DMEM after AICAR led to no significant change in activity compared to that of untreated cells (Figure 2C–E). These data suggest that the presence of nutrients in DMEM modulates the downstream metabolic response to AICAR following recovery. 

**Figure 3:**
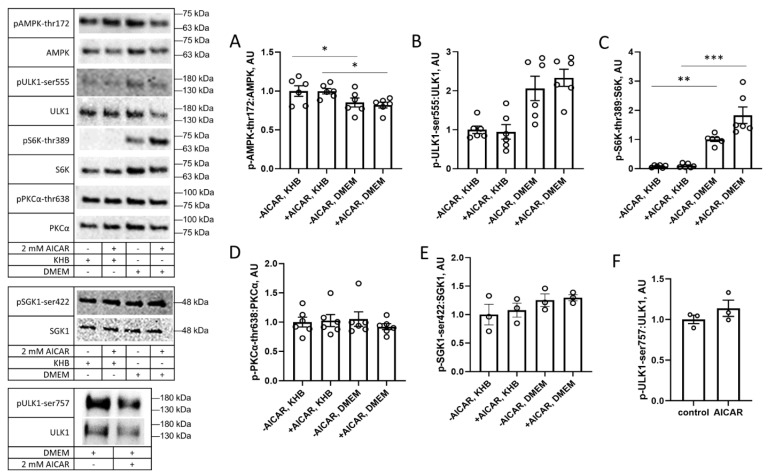
Nutrient-rich conditions support preferential phosphorylation of the mTORC1 target S6K after prior exposure to AICAR. C2C12 cells were treated with 2 mM AICAR for 1 h; AICAR was then washed out and cells incubated in DMEM or KHB for three hours before protein harvest. Phosphorylation of (**A**) AMPK, (**B**) AMPK target site on ULK1, and mTOR target sites on (**C**) S6K, (**D**) PKCα, (**E**) SGK1, and (**F**) ULK1, was assessed by Western blot. Values are means ± SEMs. *n* = 6/group (*n* = 3/group for *p*: total SGK1-ser422 and *p*: total ULK1-ser757). *, *p* < 0.05; **, *p* < 0.01; and ***, *p* < 0.001. AU: arbitrary units.

The nutrient-dependent effects of prior exposure to AICAR shown in Figure 2 led to the question of whether the key nutrient sensor mTOR could also play a role in these differential effects. We tested this through assessing the phosphorylation of AMPK and its downstream substrate p-ULK1 ser555 under the presence or absence of amino acids that are known to activate mTORC1, as well as the phosphorylation of mTOR substrates. While we observed that AICAR treatment acutely increased the thr172 AMPK phosphorylation, there was no discernable difference after three-hour recovery, and we found that recovery in DMEM led to slightly reduced AMPK phosphorylation compared to recovery in KHB (Figure 3A). ULK1 phosphorylation at its ser555 AMPK target site was also unchanged by prior AICAR treatment, but the cells that recovered in DMEM, interestingly, had increased ULK1 phosphorylation compared to that of the cells that recovered in KHB (Figure 3B). Prior exposure to AICAR increased the phosphorylation of S6K at its mTORC1 target site when the cells recovered in DMEM, but not in KHB (Figure 3C). This increase in S6K phosphorylation after recovery from AICAR exposure marked a key contrast to the reported acute effects of AICAR in decreasing S6K phosphorylation [5]. 

Given these findings, subsequent experiments were performed for cells that recovered in the presence of amino acids (i.e., DMEM) versus their absence (i.e., KHB supplemented with glucose). When recovering in DMEM, prior AICAR exposure did not alter the phosphorylation of PKCα, SGK1, or ULK1 at their mTOR target sites (Figure 3D–F). Collectively, these data suggest that prior exposure to AICAR preferentially increases the mTORC1 phosphorylation of S6K instead of increasing the phosphorylation of mTORC2 targets, suggesting that the former complex may be involved in these nutrient-dependent differences. 

**Figure 4:**
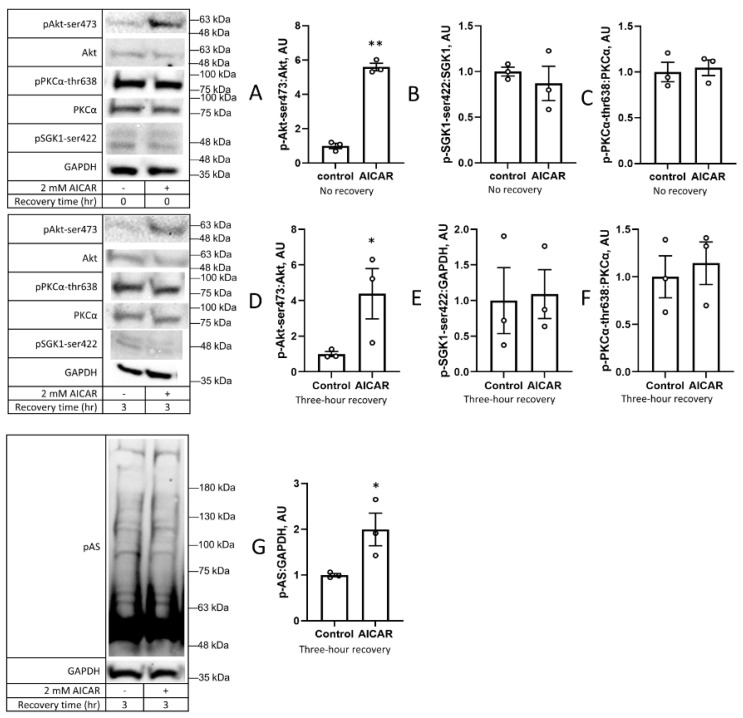
Prior exposure to AICAR alters phosphorylation of Akt at serine 473 but not other mTORC2 substrates. C2C12 cells were treated with 2 mM AICAR for 1 h, after which AICAR was removed, and either harvested immediately (**A**–**C**) or recovered in DMEM for three hours before harvesting (**D**–**G**). Akt-ser473 (**A**,**D**), SGK1-ser422 (**B**,**E**), and PKCα-thr638 (**C**,**F**) phosphorylation was measured using Western blot. (**G**) shows phosphorylation of Akt substrates (pAS) across a range of molecular weights. Values are means ± SEMs. *n* = 3/group. *, *p* < 0.05; **, *p* < 0.01. AU: arbitrary units.

Prior exposure to AICAR also caused an increase in the phosphorylation of Akt at ser-473, an mTORC2 target site. This effect appeared to be specific to Akt ser-473, as the phosphorylation of the mTORC2 targets PKCα thr-638 and SGK1 ser-422 was unchanged by prior exposure to AICAR (Figure 4). These results were consistent both immediately following the AICAR treatment and after a three-hour recovery period, indicating that this was a persistent effect due to the AICAR treatment and not due to a delayed downstream response. In parallel with the increased Akt phosphorylation after exposure to AICAR (also see Kazyken et al. 2019) [23], the phosphorylation of Akt substrates (pAS) was increased at sites matching the Akt target motif (Figure 4G). Taken together, these data suggest that prior exposure to AICAR increases the phosphorylation of Akt at its mTORC2 target site and S6K at its mTORC1 target site without altering the phosphorylation of other representative mTOR substrates (i.e., PKCα and SGK1).

**Figure 5:**
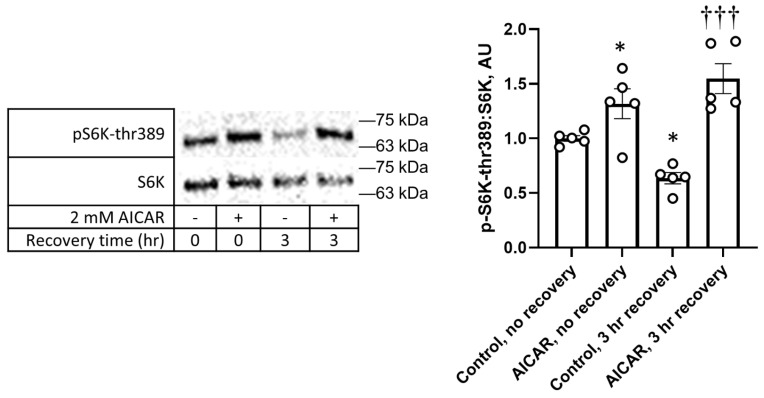
Phosphorylation of mTORC1 substrate S6K at thr389 increased with AICAR treatment in DMEM even after prolonged recovery. C2C12 cells were treated with 2 mM AICAR for 1 h and either immediately lysed and harvested or washed and recovered in DMEM for 3 h before harvest. WCL samples were tested for phosphorylation of mTORC1 target S6K-thr389 using Western blot. Values are means +/− SEMs. *n* = 5/group. *, *p* < 0.05 compared to control; †††, *p* < 0.001 compared to control, 3 h recovery. AU: arbitrary units.

Considering the immediate increase in ULK1-ser555 phosphorylation following the AICAR treatment (Figure 1C) and prolonged increase in S6K-thr389 phosphorylation following the AICAR treatment (Figure 3C), we considered whether mTORC1 activation was elevated immediately following AICAR. While we observed no change in the mTORC1 substrate ULK1-ser757 phosphorylation after three-hour recovery (Figure 3F), we observed a decrease in S6K phosphorylation before and after three-hour recovery without AICAR, and increased S6K-thr389 phosphorylation after recovering in DMEM following the AICAR treatment (Figure 5). The increase due to AICAR was also the case immediately after the one-hour treatment, suggesting a complex model of signaling, in which S6K-thr389 (but not other mTOR substrates) are phosphorylated both immediately after AICAR treatment and a prolonged recovery in C2C12 cells.

**Figure 6:**
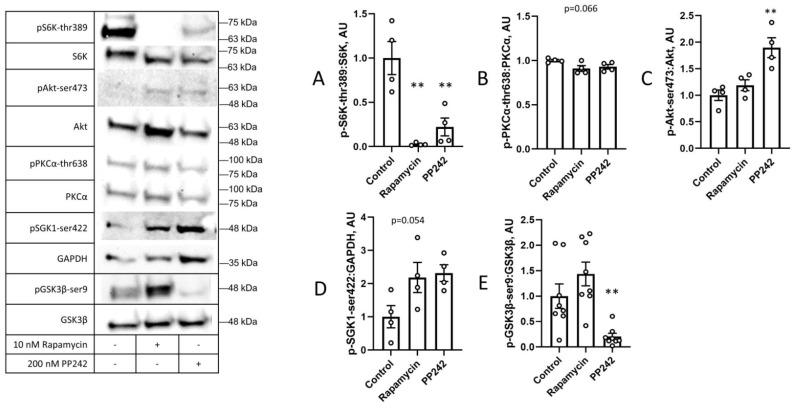
mTOR inhibitors reveal paradoxical phosphorylation of mTOR targets. C2C12 cells were treated with DMSO, 10 nM rapamycin, or 200 nM PP242 for 1 h before cells were lysed and harvested. WCL samples were tested for phosphorylation of (**A**) mTORC1 target S6K-thr389, mTORC2 targets (**B**) PKCα-thr638, (**C**) Akt-ser473, (**D**) SGK1-ser422, and (**E**) Akt target GSK3β-ser9 using Western blot. Values are means +/− SEMs. *n* = 4/group (*n* = 8/group for *p*: total GSK3β). **, *p* < 0.01. AU: arbitrary units.

The data regarding the specific increase in phosphorylation at a subset of mTOR targets raised the question of whether other kinases could be acting at these sites *in lieu* of mTOR. To address this possibility, we assessed the phosphorylation patterns after exposure to rapamycin, which inhibits mTORC1 but not mTORC2 [24], and PP242, which inhibits both mTORC1 and mTORC2 [25]. As expected, both inhibitors suppressed the phosphorylation of S6K (Figure 6A). Both mTOR inhibitors tended to suppress the phosphorylation of PKCα at its mTORC2 site (Figure 6B). Strikingly, PP242 increased the Akt phosphorylation at its mTORC2 site (Figure 6C). Both rapamycin and PP242 tended to increase the phosphorylation of SGK1 at its mTORC2 site (Figure 6D). Paradoxically, PP242, which increased the Akt phosphorylation two-fold, almost completely eliminated the GSK3β phosphorylation at its Akt target site, ser9 (Figure 6E). Taken together, these data suggest the complex regulation of the phosphorylation of mTOR targets. In particular, the data suggest that either a kinase other than mTORC2 participates in Akt-ser473 phosphorylation, or mTOR regulates phosphatases that act on its substrates.

## 4. Discussion

The new information provided by this study shows that prior treatment with AICAR in differentiated C2C12 cells led to a selective increase in the phosphorylation of the mTORC1 target S6K and the mTORC2 target Akt, despite the phosphorylation of AMPK returning to baseline following recovery. Further, the C2C12 cell response to prior treatment with AICAR appeared to be dependent on the presence or absence of nutrients, in particular amino acids. It should be noted, however, that due to the density of the cells studied, most but not all of the myocytes were differentiated at the time of experimentation, and therefore the results described in this report do not exclusively refer to myotubes. Another caveat is that this was an in vitro study in which cell cultures were used to mimic myofibers. Although beyond the scope of this study, further ex vivo studies on harvested tissue lysates and sections would add additional perspectives to our findings.

In a nutrient-replete state, such as after eating a carbohydrate-rich meal, the pancreas secretes insulin, which binds to insulin receptors on the cell membrane, activating insulin receptor substrate 1 (IRS1)-based signaling that activates phosphatidylinositol-3-kinase (PI3K) [26]. PI3K generates phosphatidylinositol-3,4,5-phosphate (PIP3) and is an activator of PI3K-dependent kinase 1 (PDK1). The former localizes protein kinase B (also known as Akt) to the plasma membrane, where it is phosphorylated by PDK1 and a mammalian target of rapamycin (mTOR) complex 2 at thr-308 and ser-473, respectively [27,28]. Both phosphorylation events activate Akt, which enables it to phosphorylate the Akt substrate of 160 kDa (AS160), a Rab GAP that regulates the vesicles containing the glucose transporter GLUT4 [29,30]. The phosphorylation of AS160 leads to its inactivation, resulting in GLUT4 translocation to the cell membrane and increased glucose transport in skeletal muscle cells [31]. While AMPK phosphorylation and activity return to basal levels by three hours post-exercise [14,15] (Figure 3A), the exercise-induced phosphorylation of Akt substrates such as AS160 persists for several hours post-exercise [32]. 

In the current study, increased Akt substrate phosphorylation after prior exposure to AICAR (Figure 4G) was associated with increased Akt phosphorylation at its mTORC2 target site, ser-473 (Figure 4A,D). In addition to Akt, mTORC2 has several other downstream effectors that are involved in cytoskeletal restructuring, including serum and glucocorticoid kinase 1 (SGK1) [33] and protein kinase C alpha (PKCα) [34]. However, we did not see a change in their phosphorylation statuses, either immediately after AICAR treatment or after a three-hour recovery period (Figure 3D,E; Figure 4B,C,E,F). These suggest a nuanced phosphorylation profile of mTORC2 substrates following AICAR treatment and, crucially, after a prolonged recovery period in DMEM following treatment.

While mTORC2 aids in the Akt-induced phosphorylation of AS160 [30], mTORC1 is activated in nutrient-replete cells and promotes cell proliferation and protein synthesis. Protein synthesis is regulated by mTORC1 through the phosphorylation of ribosomal protein S6 kinase (S6K) and eukaryotic translation initiation factor 4E binding protein (4E-BP1) [35]. Additionally, mTORC1 also phosphorylates ULK1 at its ser-757 site directly and GSK3β at its ser-9 site indirectly via Akt activation, inhibiting both [36,37]. Despite elevated S6K phosphorylation after a three-hour recovery period in DMEM (Figure 5), we found that the ULK1-ser757 phosphorylation was unchanged (Figure 3F). A similar paradox was reported when a 1 h bout of cycling exercise increased the serine 2448 phosphorylation of mTOR, a marker of mTORC1 activity, yet did not alter the pULK1-ser757 levels [38]. Since AMPK phosphorylates ULK1 at ser-555 (an event that activates it [36]), perhaps this causes a conformational change that stabilizes it and prevents it from being phosphorylated at ser-757, even after AMPK activity has returned to baseline. This could explain why, despite elevated S6K phosphorylation both immediately and after a three-hour recovery period (Figure 5), the p-ULK-ser757 levels did not increase following recovery, as would be expected with mTORC1 activation.

Both mTOR complexes are similar, with the primary distinction being the presence of either Raptor (in mTORC1) or Rictor (in mTORC2) [39]. They are also believed to be mutually exclusive: the dissociation of one complex leads to the formation of the other, depending on the circumstances [28]. In the current study, the prior exposure of C2C12 cells to AICAR led to S6K phosphorylation when the cells recovered in DMEM, but not in KHB containing glucose (Figure 3C). Presumably, this was an effect of amino acid availability that could activate mTORC1 via the effects of cytosolic amino acids, including leucine, arginine, and glutamine [7]. Recovery in DMEM also prevented prolonged decreases in glycolytic activity, which indicated that the changes to ULK1 and subsequent cell catabolism were due to AMPK-activated signaling and not a general effect of the AICAR metabolite ZMP on glycolysis (Figure 2).

Autophagy, a self-degradative cellular process, is highly upregulated in response to cell stress, the excess accumulation of proteins, or low nutrient resources [40]. What results is the degradation of misfolded or accumulated protein aggregates, as well as the generation of substrates for glycolysis [41], protein synthesis [42], and antioxidant activity [43]. The autophagy initiation complex contains unc-like protein kinase 1 (ULK1), a key metabolic sensor that is regulated by cell nutrient status and is required for functional autophagy [44]. In the current study, the phosphorylation of ULK1 at its AMPK target site was elevated immediately following the AICAR treatment, but returned to baseline after three-hour recovery (Figure 1C). What is especially intriguing is that, despite recovery in DMEM leading to the increased phosphorylation of the mTORC1 substrate S6K, the ULK1 phosphorylation at its mTORC1 target site (ser757) was unchanged following the AICAR treatment (Figure 3E), and the phosphorylation at its AMPK target site (ser555) was increased when recovered in DMEM compared to KHB (Figure 3B). Sustained ULK1 phosphorylation might possibly play a role in increased Akt phosphorylation as well, as ULK1 activation from mTORC1 inhibition may initiate the activation of the PI3K Vps34 downstream, resulting in the generation of PI3P, the activation of PDK1, and the phosphorylation of Akt at thr308 [45]. This then primes Akt for phosphorylation full activation via phosphorylation at its mTORC2 target site, ser473 [46,47]. This pathway, alongside the data on other mTOR substrates, paints a heterogenous profile of mTOR substrate phosphorylation after AICAR treatment followed by recovery in amino-acid-rich DMEM.

PP242 inhibits mTOR activity by binding at the ATP-binding site, resulting in decreases in the activity of both mTOR complexes [48]. Since the serine 473 phosphorylation of Akt is a major action of mTORC2 [49], we expected that the inhibition of mTORC2 would lead to a decrease in the phosphorylation of Akt-ser 473 and other mTORC2 substrates such as SGK1-ser422 and pPKCα-thr638. We instead saw an increase in the pAkt-ser473 levels, a trend towards an increase in pSGK1-ser422, no change in pPKCα-thr638, and a decrease in pGSK3β-ser9. PP242 has an IC_50_ of 8 nM and is highly specific, its next-closest inhibition target being DNA-dependent protein kinases at 410 nM [48,50,51]. Our dose of 200 nM, therefore, would be high enough to stymie mTOR activation without substantially inhibiting other similar kinases. This suggests that there is a different pathway through which Akt is phosphorylated at this site (Figure 6C); the proposed candidates include mitogen-activated protein kinase-activated protein kinase-2, integrin-linked kinase, p38, PKCβ, NIMA-related kinase, double-stranded DNA-dependent protein kinase, and ataxia telangiectasia mutated [52].

In conclusion, prior exposure to the AMP analog AICAR caused a unique downstream phosphorylation profile in C2C12 cells immediately following and after a sustained recovery period. Under nutrient-replete conditions, prior exposure to AICAR caused a prolonged increase in the phosphorylation of the mTORC1 and mTORC2 targets, S6K and Akt, respectively. However, the phosphorylation of the other mTORC1 and mTORC2 targets, ULK1, SGK1, and PKCα, was unchanged. Thus, metabolic stress simulated by AICAR was sufficient to activate mTOR-regulated pathways, leading to protein synthesis (S6K) and the acquisition and storage of nutrients (Akt).

## Data Availability

The raw data supporting the conclusions of this article will be made available by the authors with no undue reservation.
